# Supplementary material for: Identification of photoperiod-induced specific miRNAs in the adrenal glands of Sunite sheep (Ovis aries)
Source: Front Vet Sci. 2022 Jul 22;9:888207. doi: 10.3389/fvets.2022.888207 (PMC9354845; doi:10.3389/fvets.2022.888207)
Supplement: Supplementary file 1 [file Data_Sheet_1.ZIP › Supplementary file/miRDeep2 file.zip!/miRDeep2 file/Know_Structure/SP42A2/oar-mir-431.pdf]

miRBase precursor	: oar-mir-431
Total read count	: 260
oar-miR-431 read count	: 46
remaining reads	: 214

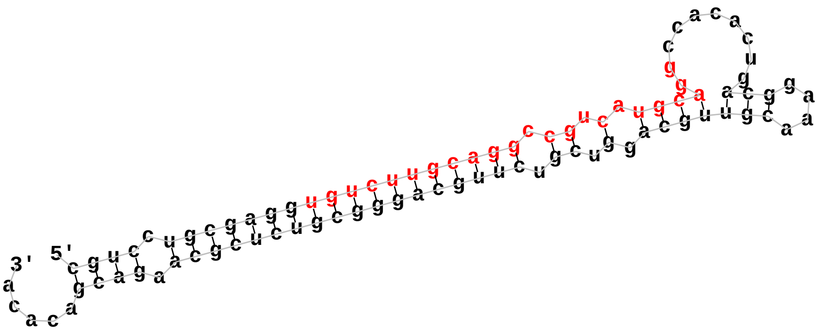

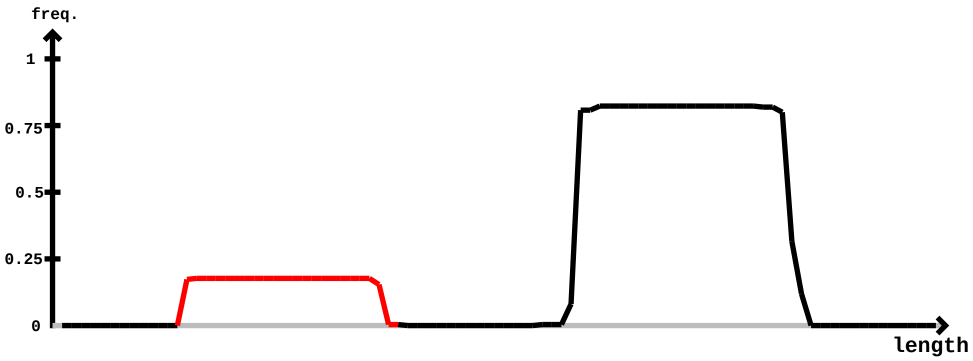


```
                oar-miR-431
5'- cguccugcgaggugucuugcaggccgucaugcaggccacacugacggaaacguugcaggucgucuugcagggcgucucgcaagacgacaca  -3'   exp
    ((((.((((((((((((((((((.((.(.((((..........(((....))))))).).)).)))))))))))))))))).)))).....     reads      mm        sample
    ............ugucuugcaggccgucaugc...........................................................     5          0         seq
    ............ugucuugcaggccgucaugca..........................................................     39         0         seq
    ............ugucuugcaggccgucaugcagg........................................................     1          0         seq
    .............gucuugcaggccgucaugc...........................................................     1          0         seq
    .................................................acguugcaggucgucuugcagggcguc...............     1          0         seq
    ....................................................uugcaggucgucuugcagggcgu................     12         0         seq
    ....................................................uugcaggucgucuugcagggcguc...............     4          0         seq
    ....................................................uugcaggucgucuugcagggcgucu..............     4          0         seq
    .....................................................ugcaggucgucuugcaggg...................     1          0         seq
    .....................................................ugcaggucgucuugcagggcg.................     5          0         seq
    .....................................................ugcaggucgucuugcagggcgu................     112        0         seq
    .....................................................ugcaggucgucuugcagggcguc...............     44         0         seq
    .....................................................ugcaggucgucuugcagggcgucu..............     27         0         seq
    .......................................................caggucgucuugcagggcgu................     2          0         seq
    .......................................................caggucgucuugcagggcguc...............     2          0         seq
```
